# Functional myelin in cognition and neurodevelopmental disorders

**DOI:** 10.1007/s00018-024-05222-2

**Published:** 2024-04-13

**Authors:** Hasni Khelfaoui, Cristobal Ibaceta-Gonzalez, Maria Cecilia Angulo

**Affiliations:** 1 Université Paris Cité, Institute of Psychiatry and Neuroscience of Paris (IPNP), INSERM U1266, 75014 Paris, France; 2 GHU-PARIS Psychiatrie Et Neurosciences, Hôpital Sainte Anne, 75014 Paris, France

**Keywords:** Myelin, Cognition, Oligodendrocytes, OPCs, Development, Schizophrenia, Autism spectrum disorder, Brain oscillations

## Abstract

In vertebrates, oligodendrocytes (OLs) are glial cells of the central nervous system (CNS) responsible for the formation of the myelin sheath that surrounds the axons of neurons. The myelin sheath plays a crucial role in the transmission of neuronal information by promoting the rapid saltatory conduction of action potentials and providing neurons with structural and metabolic support. Saltatory conduction, first described in the peripheral nervous system (PNS), is now generally recognized as a universal evolutionary innovation to respond quickly to the environment: myelin helps us think and act fast. Nevertheless, the role of myelin in the central nervous system, especially in the brain, may not be primarily focused on accelerating conduction speed but rather on ensuring precision. Its principal function could be to coordinate various neuronal networks, promoting their synchronization through oscillations (or rhythms) relevant for specific information processing tasks. Interestingly, myelin has been directly involved in different types of cognitive processes relying on brain oscillations, and myelin plasticity is currently considered to be part of the fundamental mechanisms for memory formation and maintenance. However, despite ample evidence showing the involvement of myelin in cognition and neurodevelopmental disorders characterized by cognitive impairments, the link between myelin, brain oscillations, cognition and disease is not yet fully understood. In this review, we aim to highlight what is known and what remains to be explored to understand the role of myelin in high order brain processes.

## Introduction

In the central nervous system (CNS), oligodendrocytes (OLs) are responsible for the production and maintenance of the myelin sheath. OLs originate from oligodendrocyte precursor cells (OPCs), which arise in the embryonic (ventral OPCs) and perinatal (dorsal OPCs) mouse telencephalon in distinct successive waves [1, 2]. OPCs also persist as a major pool of progenitors in the adult brain long after oligodendrogenesis completion and promote remyelination when necessary [3–9]. However, OPCs should not be constrained to their OL progenitor function as they are arising as circuit regulators in the parenchyma with functions ranging from neuronal migration to glial scar formation ([10] extensively reviews these non-canonical functions). Along the same line, an extensive body of evidence links OLs to a variety of roles such as energy metabolism, neuroprotection, axonal maintenance and information processing [11–14]. Although OLs and the myelin sheaths they generate are considered a single cellular entity, functional studies often discriminate between roles strictly related to the cells themselves and those specifically associated with myelin per se.

**Fig. 1:**
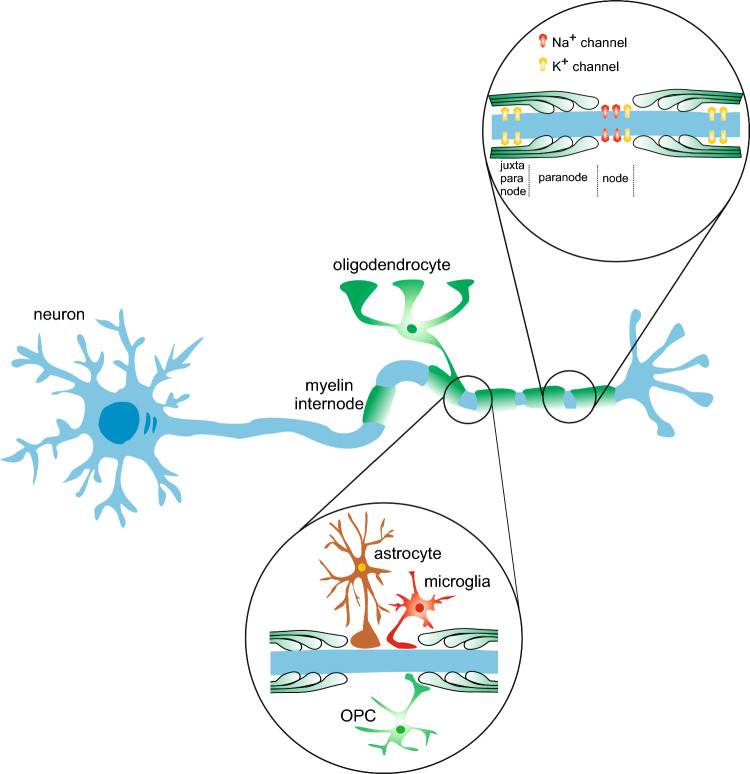
Myelin sheath, nodal architecture and axo-glial contacts. Axons bear multiple myelin sheaths originating from mature oligodendrocytes and consisting of the internode, juxtaparanodes and the paranodes which directly flank the nodes of Ranvier (top). Sheath architecture plays a crucial role in the segregation of nodal proteins, especially voltage-gated Na^+^ and K^+^ channels, participating in the generation of a nodal structure that is essential to action potential conduction and maintenance. Finally, the NORs are also contacted by other glial cells such as astrocytes, microglia and OPCs (bottom). They constitute a dynamic region of the axolemma and a site of modulations that serve many functional and structural purposes

The myelin sheath is a highly specialized multilamellar membrane that wraps axons. Central myelin is structured by an ensemble of compact interconnected lamellae of membrane that contact the axon through terminal loops forming the axo-glial paranodal junction directly followed by the juxtaparanode located beneath the compacted myelin of the internode [15, 16]. What we usually refer to as the myelin sheath is the single entity formed by the paranodes, juxtaparanodes and the internode (Fig. 1). Adjacent myelin sheaths are separated by nodes of Ranvier (NORs) [17], unmyelinated regions where the nerve fiber is laid bare and frequently contacted by other glial cells including astrocytes [18, 19], OPCs that contact both the node and paranodal myelin and whose role is yet to be resolved [20] and finally, microglia that could play a role in remyelination when myelin sheath integrity is impaired [21, 22] (Fig. 1). Myelin’s high compartmentalization allows a distinctive sheath-axon interaction that serves various functional ends such as the distribution of glial metabolites [16, 23]. In intimate relationship with the unmyelinated segments or NORs, the myelin sheath enables action potential regeneration and propagation along fibers of varying lengths, resulting in saltatory conduction, an energy-, space- and time-saving phenomenon.

The function of myelin can be approached at two different levels: at the local level, which consists of insulating axons, supporting them and ensuring bidirectional axo-glial communication, and at the global level, which orchestrates the interconnection of neuronal assemblies and the association of functional brain hubs for complex information processing. In this review, we will first introduce some general concepts about the role of myelin in action potential conduction and axonal metabolism (local level). Next, we will discuss how myelin partakes in cognitive processes and assess whether myelination might modulate neuronal network activity and cortical oscillations during the execution of these processes (global level). Finally, we will address the relevance of myelin defects in neurodevelopmental disorders (NDDs) associated with cognitive deficiencies.

## Generalities on myelin in conduction and metabolic coupling

Saltatory conduction has long been held as the main phenomenon resulting from myelin sheath wrapping around electrically active axons [24, 25]. In electrophysiological terms, myelin works such wonders by decreasing the capacitance of the axolemma while increasing resistance to ion flow. This achieves two important features: (1) conduction speed of action potentials is increased while (2) their electrical properties are maintained throughout the traveled distance. Beyond physical insulation of the axon, the segregation of crucial proteins by the various segments of the sheath directly impacts conduction. Voltage-gated Na^+^ channels (Na_V_ channels) are exclusively stabilized at the NORs by the paranodal axo-glial junction [26, 27] (Fig. 1). In this small, well-defined region of the axon, an increase in membrane potential up to the threshold potential induces Na_V_ channels opening, generating the strong depolarization (or rising phase) characteristic of the action potential. Na_V_ channels are expressed throughout the brain but their isoform combinations, density and pattern of expression differ in GABAergic interneurons as compared to glutamatergic neurons due to the morphological and functional differences of these two populations. A significant distinction between the two cell types in the rodent cerebral cortex is that GABAergic interneurons primarily express Na_V_1.1, while glutamatergic neurons predominantly express Na_V_1.2 and Na_V_1.6. Additional differences also emerge during development and vary across species [28, 29]. During action potential generation, Na_V_ channels rapidly inactivate allowing K^+^ channels to repolarize the axon. Different voltage-gated K^+^ channels as well as mechano- and thermo-sensitive K^+^ channels, two-pore-domain potassium (K2P) channels, actively drive repolarization (or falling phase) at the nodes [30–32], wherein K_v_1 channels located in the juxtaparanode contribute to the refractory period [33, 34] (Fig. 1). Similar to Na_V_ channels, the expression patterns of K^+^ channels may vary depending on the developmental stage and specific cell types. While certain channels, such as K_v_1 and K_v_7, can be expressed on the axons of both glutamatergic and GABAergic neurons [34, 35], K_v_3 channels are particularly suited to supporting the high-frequency repetitive firing characteristic of GABAergic fast-spiking interneurons (although their expression is not solely associated with this function) [36]. Regarding K^+^ homeostasis, OL express inwardly rectifying K^+^ channels (K_ir_ channels) actively participating in K^+^ buffering and contributing to OL-axon metabolic coupling [37, 38]. They also drive the establishment of axolemma resting potential allowing OLs to indirectly adjust neuronal excitability, a phenomenon especially relevant in white matter tracts where astrocytes, the main actors of extracellular potassium uptake, have limited access to axons [39]. Beyond action potential propagation at the nodal axolemma, the myelin sheath also forms a singular structure with the insulated internodal axolemma and appears to generate potentials through the periaxonal and paranodal domains thus forming another axonal conducting pathway outside of NORs. This phenomenon, termed “submyelin conduction”, could play a role in the spatiotemporal profile of action potential saltation [40], further supporting the notion of an electrophysiological coupling between the axon and its myelin sheaths.

As well as promoting fast conduction, myelin sheaths have emerged as a central component of axon metabolism [14]. Axonal access to energy-rich extracellular metabolites is limited by myelin insulation, so it is the myelin itself that has to supply the energy. Reminiscent of the astrocytic “lactate shuttle”, OLs transport lactate, a product of aerobic glycolysis, through the periaxonal space into the axon via a pair of specialized monocarboxylate transporters (glial MCT1 and axonal MCT2) allowing a myelin-axon metabolic crosstalk that is relevant in both health and disease [14, 41, 42]. This axo-glial coupling is highly plastic and follows axon energy needs through the retroactive action of axons on their myelin sheath. Notably, neuronal activity probably increases glutamate levels in the periaxonal space and activates NMDA receptors expressed in the paranodal and internodal membranes of OLs, enabling them to tune their energy production to neuronal activation [43, 44]. Beyond energy provision, myelinating cells secrete various neuroprotective and neuro-supportive factors into the periaxonal space. For instance, glutamate activation of glial NMDA receptors triggers the secretion by OLs of exosomes carrying specific protein and RNA cargos that are endocytosed by neurons, improving oxidative stress resistance and long-term axonal integrity maintenance [45, 46]. Although the nature of these cargos and the signaling cascades they elicit in the axon remain largely unknown, some have been identified such as ferritin heavy chain (FTH1), a strong iron chelator protein that is secreted by OLs into the adjacent extracellular space to prevent ferroptosis, the accumulation of free iron ions that generates harmful oxidizing species [47]. Although the functional and metabolic coupling between myelin sheaths and axons is important for the dynamics of myelin-axon interactions, its significance goes far beyond local crosstalk, as it determines the action potential waveform and affects neuronal coding and activity in a network, ultimately influencing information processing in the brain. Over the last decade, myelin has emerged as an important component of plasticity, memory and learning, moving away from its primary function associated with the transmission and velocity of action potentials.

## Myelination heterogeneity in the cortex

**Fig. 2:**
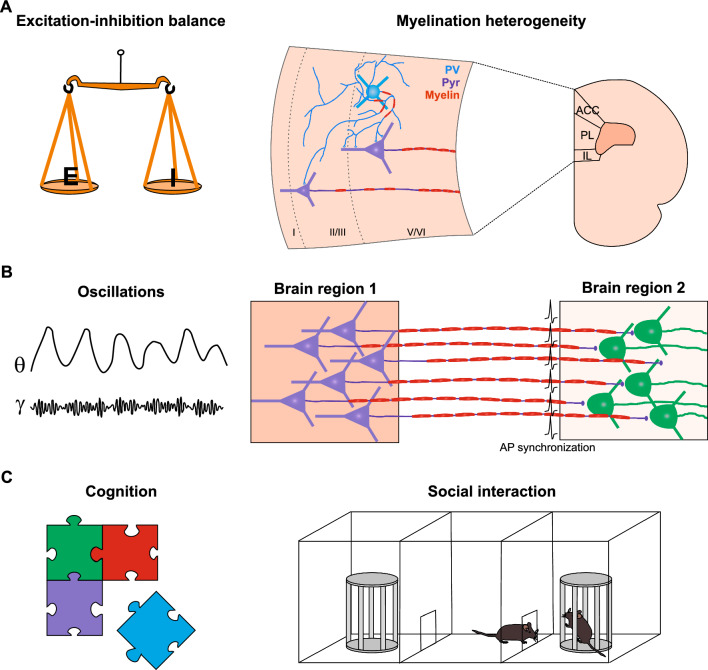
Summary of myelination effects at cellular, network and cognitive levels.** A** Myelination patterns (myelin in red) of pyramidal neurons (pyr, purple) and PV interneurons (PV, blue) are different in the cortex, probably including the mPFC (right). It has been shown that myelination can affect the excitation-inhibition balance (left, [65]). However, it is unknown how myelination heterogeneity impacts conduction and synaptic transmission. IL and PL: infralimbic and prelimbic regions of the mPFC; ACC: anterior cingulate. **B** Myelin may adjust the synchrony between neuronal ensembles of two distant brain regions (right). The synchrony of local (intracortical) and inter-regional neuronal networks generates brain oscillations at different frequencies such as gamma and theta, respectively (left). **C** Myelin appears to be important for the proper performance of cognitive tasks such as social interactions. Behavioural performance also highly depends on brain oscillations which, in turn, are influenced by myelination

The cortex contains a patchwork of differentially myelinated axons belonging to both glutamatergic and GABAergic neurons. Presence and extent of myelination relies in part on axon diameter, as OLs tend to extensively myelinate larger axons [48, 49]. However, complex patterns of myelination are also present in sub-diameter threshold axons while some axons with the required diameter are discontinuously myelinated or not myelinated at all. Beyond axonal diameter, other factors could be driving myelination heterogeneity, notably in gray matter: OL intrinsic properties [50], axonal permissive cues [51] and somatodendritic repulsive cues [52], neuronal activity [53–58], as well as localization and cell type [59, 60]. Comparing GABAergic and glutamatergic neurons has revealed compelling differences in myelin content and patterns. On one hand, high throughput electron microscopy that individually traces pyramidal cell proximal axons in the mouse somatosensory cortex uncovered a myelin gradient, with deep layer (V/VI) pyramidal cells displaying a higher myelin coverage compared to superficial layer (II/III) pyramidal neurons. Furthermore, superficial layer pyramidal cells display a distinct longitudinal myelin pattern with myelin sheaths being separated by long unmyelinated gaps (much longer than NORs) (Fig. 2A, right; [60]). On the other hand, a vast majority of myelinated gray matter GABAergic axons belong to parvalbumin-expressing neurons (PV interneurons) [59, 61, 62]. Moreover, the extent of PV interneuron myelination appears to be proportionally scaled to the overall myelin content across different cortical areas [63]. PV interneuron myelin topography is dictated by axonal diameter and interbranch distances in both human and rodent neocortex [64]. However, this myelination is limited to the proximal part of the axon (< 3%), so that most of the axon is free of myelin [59], suggesting the existence of other subtle interactions between OLs and specific axonal regions (Fig. 1A, right). As PV interneurons have only short local projections, their myelination was at first quite puzzling but has since been shown to serve a cardinal role in their functional maturation. Indeed, an early disruption of PV interneuron-OPC synapses results in a dysmyelination and an abnormal proximal axon morphology. These defects are associated with a decrease of their high firing frequency and a disturbance of their synaptic connectivity that reduces the inhibitory drive of the somatosensory cortex, particularly in layer IV [65]. Furthermore, impairing myelination early in development (as early as postnatal day 21 in mice) reduces their autaptic responses, i.e. a reduction in functional GABAergic synapses of the PV interneuron onto itself. In turn, this absence of autaptic control results in an exacerbated excitability of PV interneurons, which become unable to sustain optimal high frequency firing rates [66]. Another compelling function of PV interneuron myelin sheaths is the clustering of mitochondria and thus the fine tuning of metabolic requirements during axonal activation [67]. Interestingly, other GABAergic neuron subtypes such as a fraction of somatostatin (SST)-expressing interneurons and, to a much lesser extent, vasoactive intestinal peptide (VIP)-expressing interneurons have myelin sheaths with layer-specific arrangements [63, 68, 69]. However, the functional and structural significance of this myelination, sometimes limited to a single internode per axon, remains largely unexplored, pointing to avenues for further investigation. Nevertheless, it has recently been reported that a myelin loss in SST interneurons in the hippocampus can be associated with a reduction in their firing frequency [69]. Finally, comparisons between seven different GABAergic and glutamatergic neurons show significant variations in their myelination patterns, with PV interneurons and thalamocortical axons exhibiting higher myelination probabilities [63]. The cell identity, the axon diameter and the axonal degree of branching (collateral formation) dictate the myelin profile along axons belonging to distinct neuronal subtypes [63, 64].

We would like to highlight two important ideas: (1) although white matter myelin has been extensively studied both in health and disease, we should exert caution when extending such findings to gray matter myelination which is protracted, sparse and, as shown above, has topographical and functional complexities less found in white matter tracts [70] and (2) OL lineage cells heterogeneity (thoroughly reviewed in [71]) could be an active driver of myelination heterogeneity and should be considered in future studies.

## Myelin in cognitive processing

### Myelin establishment and cognitive development

Myelination in the CNS happens in a specific spatial and temporal order in both humans and rodents, and remains plastic and adaptive throughout life [72]. Myelin maturation in the brain progresses in a protracted fashion, from caudal to rostral, so that the prefrontal cortex (PFC) in both humans and rodents is still undergoing myelination well into early adulthood [73–78]. It is thus tempting to parallel this “late” maturation of the PFC in terms of myelination with the establishment of higher cognitive functions such as self-identity, social and emotional regulation, memory, adaptive responsiveness and predictive decision making [54, 79, 80]. In fact, correlation between white matter changes and cognitive function over the course of human life establishes a link between myelin development and cognitive development. Longitudinal brain imaging studies have shown that white matter volume—reflecting the myelin content and axonal caliber [81]—has a linear increase throughout childhood and adolescence ([82] reviews such findings while discussing various myelin imaging techniques and their shortcomings). As first reported by fractional anisotropy, a measure of white matter volume in diffusion tensor imaging (DTI), an increase of myelin thickness in frontal white matter positively correlates with increased working memory scope in children [83]. This was further confirmed by probing working memory through the maturation of fronto-parietal and fronto-striatal white matter tracts from childhood to early adulthood which revealed that proper myelination of these tracts predicts future working memory capacity [84, 85]. In terms of social-emotional skills in humans, a recent study on children aged 0 to 36 months positively correlates myelination expansion in regions of the “social brain”, such as the medial PFC (mPFC), with social-emotional development [86]. Similarly, in mice sociability is related to myelination of the mPFC as shown by the deleterious effects of social isolation immediately after weaning on both adult mPFC function and myelination [87]. During development, exposure to an early stress caused by maternal separation induces a premature differentiation of OLs in the mPFC along with emotional and object recognition impairments in the adult that can be rescued by the chemogenetic activation of mPFC neurons during the two first weeks of life [88]. As for other mPFC-related behavioral outputs, studying myelin signals in adolescent healthy subjects offers a more complete (and complex) parallel between myelin content in both gray and white matter with compulsive and impulsive behaviors [89]. The expression of both behaviors was positively correlated with reduced myelin signals in frontal areas such as the lateral and medial PFC [90]. Interestingly, the correlation was more pronounced for gray matter regions, further supporting the notion of discreet differences in myelination development and macrostructure between gray and white matter areas.

Myelination is therefore a long-lasting process that represents a perfect “substrate” for the maturation and adaptability of cognitive processes. However, although myelination and cognitive development correlate, the underlying mechanisms that link these two processes are not yet fully understood. Among many of the possible mechanisms, early neuron-OL interactions may play a decisive role in both developmental myelination and neuronal maturation. In the developing neocortex, OPCs receive transient synaptic inputs from GABAergic interneurons, mainly PV interneurons, that disappear in juvenile mice [91, 92]. The genetic inactivation of these neuro-glial synapses at an early stage of postnatal development does not have a major impact on OPC proliferation and differentiation, but leads to significant defects in interneuron myelination and in the maturation of cortical inhibitory circuits, affecting sensory discrimination [62, 65]. Furthermore, early GABA_B_ receptor-mediated signaling onto OPCs induces the apoptosis of interneurons via the cytokine TWEAK pathway, resulting in proper PV interneuron cell density and myelination in the adult [93]. The specific ablation of these receptors in OPCs is associated with an excitation-inhibition imbalance in the mPFC and severe social behavior defects [93]. These studies highlight an important role of early interneuron-OPC communication in the establishment of cortical inhibitory circuits and cognitive function (Fig. 2A).

### Experience-driven myelin plasticity

Beyond development, myelin remodeling has now emerged as one of the main drivers of plasticity in the brain. Myelin establishment during critical periods of early postnatal life is indeed paramount but not immutable, as OLs drive different myelination patterns in response to neuronal activity and experience throughout an individual’s life, thus driving a dynamic and adaptive remodeling of neuronal circuits [94, 95].

Motor learning has been extensively used as a straightforward tool to question myelin remodeling in the adult brain. In a first study on new motor skills acquisition in the rodent, Magnetic Resonance Imaging (MRI) fractional anisotropy revealed that motor training induces structural changes in the white matter of the motor cortex, and these positively correlated with the rate of learning [96]. Preventing new myelin formation in the young adult (P60 and P90) by inhibiting new OLs production directly hinders new motor skill learning [97]. Myelin plasticity concerns motor axons activated by learning and proceeds in distinct steps. Amidst learning, pre-existing myelin sheaths retract, generating a new pattern of intermittent myelination. After the learning phase, new myelin sheaths are added to cover large unmyelinated gaps on the axolemma, forming regions of continuous myelination [98].

Recent data on other brain areas involving higher cognitive processing and memory also reveal that myelin plasticity is necessary for proper behavioral performances and outcomes. For instance, spatial memory consolidation during a Morris water maze task, resulting from a complex dialogue between PFC areas, such as the anterior cingulate cortex (ACC), and the hippocampus is altered when de novo myelination is prevented in the adult [99]. Along the same line, fear learning and working memory tasks (radial arm maze) appear to increase OPC proliferation and myelination in the mPFC and ACC, respectively [100, 101], while inhibition of myelin formation impairs fear memory recall [101]. Another study has reported that the deletion of the transcription factor Olig2 in OPCs inhibits myelination, thereby impairing spatial memory in young adult mice [102]. In another register, prolonged social isolation in the adult specifically induces a decrease in myelin thickness and nuclear heterochromatin in mPFC together with a social defeat phenotype, while social re-integration for four weeks resulted in a recovery of myelin transcripts and social interaction behavior [103]. Moreover, social isolation in juvenile animals drives a hypomyelination phenotype that can be reversed in the adult by re-socialization with socially housed mice, but not socially isolated mice [87, 104], further supporting a role of myelin in driving social behavior adaptability. Myelination also affects synaptic transmission and the excitation-inhibition balance [65, 105, 106], thus having a potential impact on cortical oscillations and cognition (Fig. 2; see next section).

Cognitive processing in the adult brain thus most probably relies on the interplay of existing myelin modifications in response to neuronal activity as well as new myelin formation. In turn, myelination will adjust conduction velocity resulting in differential spike-timings that could underlie dynamic neuronal processing [65, 98, 107–109]. It should be noted, however, that these modifications may involve subtle mechanisms that go beyond a simple increase or decrease in the amount of myelin, as they may primarily produce a marked change in the length of NORs. Such a change has been observed in the adult mouse brain following a repetitive transcranial magnetic stimulation or the execution of 8-arm radial arm maze task [110]. It should also be considered that it is difficult to disentangle the molecular and cellular pathways as well as the exact role of each player of the myelination process (OPC, OL or myelin) in the observed neuronal and cognitive processing alterations and that further investigation is needed.

## Brain oscillations, myelin and cognition

The cerebral cortex exhibits sustained activity, characterized by rhythms or oscillations spanning a wide frequency range from 0.05 Hz to 500 Hz [111]. Each frequency band can be modulated differently by cognitive processes. Slow oscillations (0.05 Hz to 30 Hz) involve coordinated activity of widespread neuronal ensembles, while high-frequency oscillations (30–500 Hz), like gamma oscillations (30–90 Hz), are more localized and crucial for information coding (Fig. 2B, left). In particular, gamma oscillations arise from fast synchronization of excitatory neuronal activity, modulated to a large extent by PV interneurons, acting as a pacemaker to time network discharges [111–113]. Despite variations in brain size across species, gamma rhythms maintain a consistent frequency range and are modulated by cognitive mechanisms such as attention, working memory, cognitive flexibility and social cognition (Fig. 2B and C; [114]). Furthermore, different rhythms can interact, as in the phase-amplitude coupling observed between theta and gamma rhythms which has an impact on cognitive functions like working memory [115].

As mentioned before, myelin plasticity, as another form of activity-dependent plasticity, is relevant not only to nervous system development but also to complex information processing tasks. By its capacity to speed up action potentials and mediate proper spike-timing, it has been widely assumed that myelin influences the synchronization of neuronal ensembles in the brain (Fig. 2B, right). On a broader scale, myelin architecture in humans correlates strongly with functional connectivity mediated by neuronal oscillations in the beta and low-gamma bands, reinforcing the idea of a close relationship between myelination and specific functional networks [116]. However, despite emerging evidence for the role of myelin in cognitive processes involving coupling and synchrony, only few studies have attempted to disentangle its impact on the generation and maintenance of brain oscillations. Nevertheless, myelin plasticity offers a mechanism for modifying conduction delays in an activity-dependent manner, potentially optimizing rhythmic activity in the brain [109]. Mathematical modeling and simulations have addressed this question and proposed that myelin facilitates the synchronization of axon spikes coming from distant populations of neurons whose activity is correlated [117]. The model predicts that myelin plasticity in response to local action potentials of myelinated axons adjusts the spike temporal dispersion that occurs across these individual axons, thereby optimizing the precision of axonal discharges and promoting synchrony. Although this work supports a role for myelin in the generation of cortical oscillations, the mechanism linking myelination and neuronal synchronization is probably more complex. In vivo analyses of the auditory system revealed that dysmyelination resulted in expected conduction delays and desynchronization of inputs, along with a potential misdistribution of axonal channel proteins at NORs. Interestingly, it was shown in the same study that OL-dependent metabolic deficits, independently of myelin content and other significant structural alterations in the axon, also disrupted the temporal precision of neuronal spikes, akin to those observed in dysmyelinated mice [12]. This suggests the necessity of axoglial metabolic support for temporal auditory processing. Moreover, the heterogeneity of myelination patterns, which is patchy in pyramidal cells [60] and restricted to the proximal part of the axon in PV cells of the cortex (Fig. 2A, right; [59]), probably has an impact on axonal conduction and needs to be taken into account. In another experimental study using a cuprizone-induced demyelination mouse model, Dubey et al., assessed the role of myelin in the generation of oscillations in the primary somatosensory cortex [106]. They observed that demyelination selectively amplifies theta power during periods of quiet wakefulness (but not active states) and proposed that this effect was caused, at least in part, by a decrease in the excitability of PV interneurons and fast GABAergic transmission. Furthermore, during the in vivo optogenetic stimulation of PV interneurons at a low gamma frequency of 30 Hz, local field potential recordings revealed that this stimulation entertains an oscillatory activity at this frequency in control mice. However, following demyelination, the same optogenetic stimulation did not modulate or maintain the gamma rhythm. By simultaneously recording the ACC region and the hippocampus immediately after contextual fear conditioning, Steadman et al. show that the coupling between spindle oscillations in the prefrontal cortex and sharp wave ripple oscillations in the hippocampus was increased in controls but unchanged in mice with a disrupted oligodendrogenesis [99]. These results indicate that the production of new OLs is required for learning-induced increases in coordinated hippocampal-cortical activity. However, this effect is probably not due to myelin deficiencies since it occurs just after training.

Despite sparse studies on the role of OLs and myelin in the synchronization of neuronal networks and brain rhythms, how myelin influences different brain oscillations during behavior, especially during cognitive processes, is largely underexplored. Local in vivo electrophysiological recordings in behaving mice with genetically determined or induced alterations in myelination would be necessary to unravel how myelin is involved in behaviorally modulated cortical rhythms. The detection and quantification of brain oscillations is an advanced field of neuroscience that allows multiple brain areas to be recorded simultaneously, in some cases using more than a thousand electrodes, while the animal performs a cognitive task. This type of studies represents a major challenge and a future line of research in the field of myelin.

## Myelin in neurodevelopmental diseases (NDDs)

As an early onset phenomenon with a protracted evolution throughout life, myelination has emerged as a potential key player in NDDs, disorders that have their onset during childhood and adolescence. These two critical periods of development represent sensitive time windows for environmentally induced modifications and damage to central myelin structure and functions. Here we discuss the involvement of myelin in Autism Spectrum Disorders (ASD) and schizophrenia, two major NDDs characterized by overlapping symptoms such as communication difficulties and social withdrawal.

### Autism spectrum disorders

As a classic example of NDD etiology, consisting of a mix of genetic and environmental risk factors, an accumulating body of evidence demonstrates that myelin deficits underlie altered communication between major brain hubs in ASD individuals [118]. As demonstrated by MRI and DTI, white matter disruptions are widespread in children and adolescents with ASD [119–121]. Intriguingly, the white matter of autistic patients is overgrown during the first two years of life, but tends to be smaller than controls as they age [122]. These observations in humans were recently confirmed and termed “precocious myelination” in a murine model of ASD (BTBR mice) in which the number of OLs and myelin content in the frontal brain of neonatal pups was increased [123]. This accelerated postnatal development of the brain in ASD patients that tends to normalize and worsen with age might be more nuanced for gray matter myelination. It has been shown using T1w and T2w MRI that the overall spatial patterns of intracortical myelin distribution are similar between ASD children, aged 1.5 to and 5.5 years old, compared to typically developing children, but the age-related increase in intracortical myelination is impaired in ASD children [124]. At the cellular level, OPCs cultured from a mouse model of ASD (Pten^m3m4^) have an enhanced proliferation rate and a premature maturation onto OLs [125]. This maturation defect does not lead in vivo to a greater number of OLs in the adult, as these cells die by apoptosis and produce abnormal myelin which fails to ensheath axons [125]. In another mouse model of ASD induced by the prenatal exposure to valproic acid, OL density and myelin content is decreased in adult mice in some of the main brain regions linked to social behavior, such as the mPFC, pyriform cortex and basolateral amygdala [126]. Integrated transcriptomic analyses of both ASD mouse models and ASD patients tissues further stress OL gene dysregulation and myelination defects across species, as a highlight in both syndromic and idiopathic ASD [127, 128]. Another area of interest concerns myelin proteins such as MBP, that has been put forward as targeted by an abnormal autoimmune reaction in the ASD brain [129]. However, while molecular and cellular alterations in OL biology and myelination are a hallmark of ASD, it is unknown whether these are responsible for social behavior deficits in the disease. Interestingly, frontal cortex myelin thickness reduction has been associated with a murine model of Williams syndrome (WS), a non-canonical NDD characterized by hypersociability [130], proving that conflicting behaviors, hypersociability in WS compared to hyposociability in ASD, can arise from similar myelin abnormalities (hypomyelination), further stressing the complex etiology and symptomatology of such diseases.

These studies in ASD have singled out myelin and myelinating cells as potential therapeutical targets in a few preclinical studies. Promyelinating compounds such as clemastine -which promotes OPC differentiation into OLs and has been studied as a promyelinating agent in other myelin-related disorders such multiple sclerosis [131]- appear to rescue the cellular, structural and behavioral phenotype of the ASD mouse model of Pitt-Hopkins syndrome [132], opening new and exciting areas of investigation for future therapies. Furthermore, in a mouse model of perinatal hypoxia (a condition commonly associated with ASD in humans), which exhibits significant myelination impairments, early environmental enrichment was also shown to selectively promote endogenous myelin regeneration and functional recovery in the developing white matter [133]. Therefore, an alternative therapeutic strategy to improve myelination and white matter dysfunction might lie in early behavioral intervention and environmental enrichment.

### Schizophrenia

Schizophrenia (SCZ) onset coincides with adolescence and early adulthood, but its origins can be traced back to earlier stages of development as some cognitive impairments, depression and negative symptoms can occur during childhood [134]. Many studies suggest that myelin alterations are as prevalent in this disease as they are in ASD. Although the neurobiological mechanisms underlying SCZ are not fully understood, it has been proposed that genetic and environmental risk factors during the perinatal period, either in utero or in infancy, contributes to neurodevelopmental abnormalities that may lead to impaired myelination in the adult brain [135]. The use of myelinating cells and myelin as a prism to look at this disorder is compelling because myelination is a protracted developmental process in most of the brain regions found to be dysfunctional in SCZ [80, 135]. Myelination impairments during development have been considered to result in a defective maturation of neuronal networks connectivity (the ‘dysconnectivity’ hypothesis), which could explain some of the varying cognitive symptoms in SCZ patients, including impaired cognitive flexibility [136–139]. Similarly to ASD, imaging studies questioning the structural integrity of white matter and the inter-connectivity of various brain regions have provided a better understanding of structural insults in SCZ patients [80]. Although most of the metrics used in imaging can be related to various structural components of white matter (axon diameter, fiber density, myelination), foundational work investigating both total and frontal white matter regions suggested an overall hypomyelination of the corpus callosum in human SCZ patients [140]. These findings were corroborated by post-mortem analyses of the anterior frontal cortex which showed a reduction in the expression of the two OL-associated proteins, MAG and CNPase, in SCZ patients [140]. It was subsequently observed that frontal white matter is indeed recurrently defective in chronic patients [141–143]. On the other hand, a recent study focusing on gray matter highlights more complex changes with some regions exhibiting higher and others lower myelin content in first-episode treatment-naïve SCZ patients [144].

Recent genetic, epigenetic and biochemical analyses have corroborated OL dysfunction and abnormal expression of myelin-related genes and proteins [145, 146] as well as a decrease in the density of OLs in layer V of the PFC in SCZ patients [147]. As compared to normal OPC numbers, a reduction in OL density hints at a differentiation impairment of OPCs in the PFC of these patients [148]. Moreover, SCZ-like behaviors in juvenile mice such as impaired sociability can be elicited via a DNA hypermethylation, a hallmark risk factor of SCZ, that targets genes related to OL lineage cells [149]. Overall, dysfunctional OL lineage cells could explain, to some extent, myelination insult of SCZ patients although many interrogations remain as to the origins of such disorders and their temporal unfolding, ultimately asking if dysmyelination is a cause or a result of SCZ [135, 142]. An important point of discord from human studies is the difficulty of untangling the mesh of possible myelin insults as studies include a heterogenous population of patients: chronic patients that have been medicated for years, first episode patients naïve for any treatment, high risk patients, familial genetic risk patients. They are usually age and gender matched with the controls but might still account for slight contradictory results. A standardization of patient cohorts is needed to confirm previous results and produce finer insight in the investigations. Although further research is needed on this regard, a recent report demonstrated that specific mutations in chondroitin sulfate proteoglycan 4 (CSPG4/NG2), a hallmark protein of OPCs, exhibited familial segregation in SCZ patients having significant abnormal white matter integrity [150], a finding in favor of a direct role of OL lineage cells in this disease.

All these studies stress the role of myelin in connecting functional hubs for the synchronization of distant neuronal ensembles and the production of optimal behavioral responses to a changing environment. Brain connectivity analyses indeed indicate that long-range connectivity is usually impaired in SCZ. Along with white matter integrity impairments, another substantial evidence supports a causal role of local GABAergic interneuron dysfunction in linking cortical circuit and behavioral deficits in this disorder [151]. Several reports have found that alterations in local oscillations, mainly gamma oscillations, occur during performance of cognitive control tasks [151–153]. As previously mentioned, synchronization of cortical networks in the gamma band frequency is modulated to a large extent by the activity of PV interneurons, which provide robust perisomatic inhibitory control of glutamatergic neurons [154, 155]. This probably explains why dysfunctions in PV interneurons and gamma oscillations have been associated with cognitive deficits characteristic of SCZ [151, 156, 157]. Interestingly, myelination defects occurred specifically in PV interneurons of the mPFC in a rat model displaying schizophrenia-like behaviours [158]. Considering these findings, the high levels of myelination of PV interneurons and the early and reciprocal interactions between cortical PV interneurons and OPCs, it might be possible that impairments in PV interneuron myelination compromise the integrity of precisely timed action potentials and local synchronization [159, 160]. An interesting line of study in the field of myelin will be to investigate whether PV interneuron and OL lineage abnormalities can synergize to increase the risk of developing NDDs.

Much like in ASD, myelination could be a potential biological target in SCZ. In this line, pro-myelinating drugs could be evaluated, for example, as precognitive interventions in first-episode patients. Antipsychotic drugs that could act on OL dysfunction by potentiating their differentiation and maturation, such as the NMDA receptor ligand D-serine, and lithium [161] are another area of excitement in terms of SCZ treatment, just like we previously discussed with promyelinating compounds as possible therapies for ASD.

## Data Availability

No data presented.
